# Sulforaphane as a Multi-Scale Mechano-Modulator in Cancer: An Integrative Perspective

**DOI:** 10.3390/biology15020167

**Published:** 2026-01-17

**Authors:** Xin Zhang, Lili Cheng, Yifan Han, Tailin Chen, Xinbin Zhao

**Affiliations:** 1 Key Laboratory of Biomechanics and Mechanobiology of Ministry of Education, Key Laboratory of Innovation and Transformation of Advanced Medical Devices, Ministry of Industry and Information Technology, National Medical Innovation Platform for Industry-Education Integration in Advanced Medical Devices (Interdiscipline of Medicine and Engineering), Beijing Advanced Innovation Center for Biomedical Engineering, School of Engineering Medicine, Beihang University, Beijing 100191, China; xinzhang1024@163.com (X.Z.); 20375035@buaa.edu.cn (Y.H.); 2 Hepatopancreatobiliary Center, Beijing Tsinghua Changgung Hospital, School of Clinical Medicine, Tsinghua Medicine, Tsinghua University, Beijing 102218, China; clla04628@btch.edu.cn; 3 School of Basic Medicine, Guangxi University of Chinese Medicine, Nanning 530200, China

**Keywords:** sulforaphane, anticancer, tumor biomechanics, mechanobiology, cytoskeleton, tumor microenvironment, mechanotransduction

## Abstract

Cancer progression is driven not only by biochemical signals but also by abnormal physical forces within a stiffened tumor microenvironment. This review re-examines the anticancer compound sulforaphane (SFN) through the integrative lens of tumor biomechanics. We propose SFN functions as a “mechano-modulator,” whose pleiotropic effects converge to disrupt pro-invasive mechanotransduction. SFN targets key force-sensitive pathways (e.g., YAP/TEAD, Rho/ROCK), destabilizes invasion machinery (cytoskeleton, invadopodia), and promotes tissue-level changes such as extracellular matrix remodeling. While preclinical evidence for this mechano-modulatory role is compelling, this perspective also highlights the critical need for clinical validation and discusses the key translational challenges. By systematically linking SFN’s molecular actions to the biophysics of tumor progression, this synthesis provides a novel framework for understanding its efficacy and outlines a rational path for its future development as a mechano-inspired therapeutic.

## Simple Summary

Scientists are discovering that a tumor’s physical stiffness and pressure help it grow and spread. This review looks at a natural compound from broccoli, called sulforaphane, through this new “biomechanical” lens. We suggest sulforaphane works as a “mechano-modulator”—it fights cancer not just chemically, but by helping to normalize the tumor’s distorted physical environment. By disrupting the forces and structures cancer cells use to invade, its wide range of effects can be seen as part of a single, powerful strategy. This fresh perspective could guide the development of sulforaphane as a novel, mechanics-informed approach for cancer prevention and treatment.

## 1. Introduction

Global population aging parallels a steady rise in cancer incidence, with projections estimating over 22 million new cases annually by 2035 [1]. This trend is driven by established cancer hallmarks, such as sustained proliferation, angiogenesis, and immune evasion [2,3]. Beyond these biochemical drivers, it is now clear that biophysical aberrations are equally critical [4]. Tumors develop distinct mechanical properties—including elevated stiffness, interstitial pressure, and altered cellular mechanics—that actively fuel progression, invasion, and therapy resistance [5]. Targeting this distorted mechano-environment presents a promising but underexplored therapeutic strategy, especially as conventional chemotherapy often induces severe adverse effects that compromise patient quality of life [6].

Consequently, multi-targeting natural compounds, prized for their safety and availability, have gained significant interest for cancer prevention and treatment [7]. Among these, sulforaphane (SFN)—a bioactive isothiocyanate derived from cruciferous vegetables—stands out for its broad anticancer activity [8]. SFN exhibits potent, broad-spectrum effects against various malignancies and can enhance the efficacy of conventional chemotherapeutics while mitigating their toxicities [9,10,11,12,13,14,15]. While initially studied for its ability to induce apoptosis and cell cycle arrest, more recent work highlights its potent inhibition of tumor cell migration and invasion.

This specific effect on invasion and metastasis suggests that SFN’s mechanism may extend beyond classical biochemical modulation. Cells sense and respond to mechanical cues within the stiff tumor microenvironment via mechanotransduction pathways that directly influence malignant behavior [16]. We propose that SFN’s diverse anticancer effects can be coherently interpreted through, and are potentiated by, a foundational capacity to normalize this aberrant biomechanical landscape.

In this opinion, we re-examine SFN’s anticancer portfolio through the lens of mechanobiology. We advance the hypothesis that SFN functions as a “mechano-modulator”—an agent that alters key biophysical properties (e.g., cellular traction forces, stiffness, extracellular matrix composition) that define the tissue mechanical state, thereby disrupting force-sensitive processes essential for cancer progression. This novel framework not only unifies SFN’s pleiotropic actions but also paves the way for its rational development as a mechano-inspired therapeutic strategy. This opinion also acknowledges the current limitations, including SFN’s dose-dependent toxicity, off-target effects at high concentrations exists, and the need for direct biomechanical evidence and clinical validation, which are discussed in the final outlook.

## 2. Fundamental Characteristics of SFN

### 2.1. Extraction, Bioavailability and Metabolism

**Figure 1:**
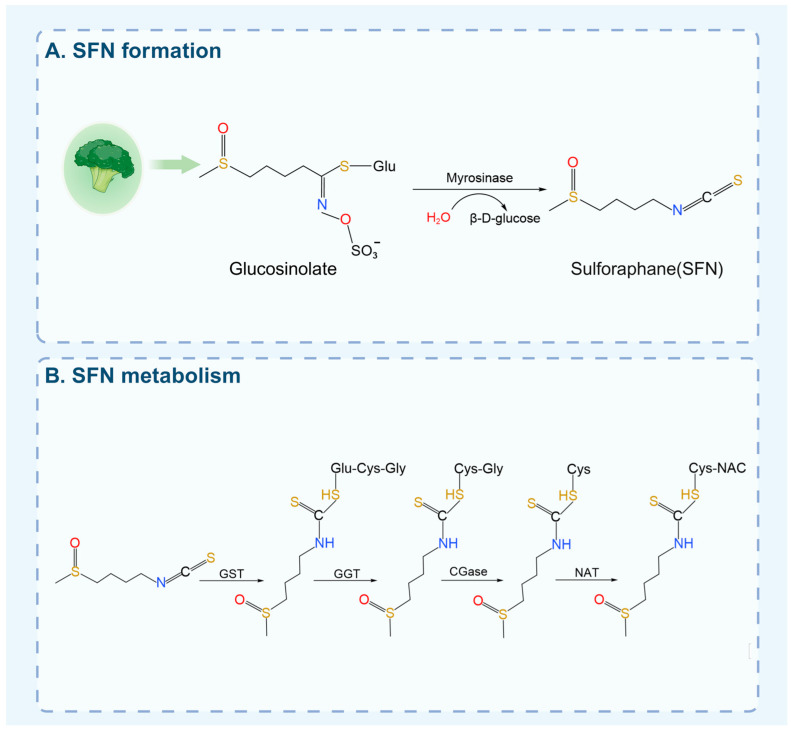
The formation and metabolic process of SFN. (**A**) SFN is generated from glucosinolates via hydrolysis by myrosinase, an enzyme activated upon plant tissue damage. Yield is influenced by source and processing, with broccoli sprouts containing significantly higher levels than mature florets. (**B**) GST catalyzes the conjugation of SFN with GSH to form SFN-GSH. This conjugate is then further processed in a stepwise manner: it is first hydrolyzed by γ-glutamyl transpeptidase to SFN-Cys-Gly, then converted to SFN-Cys by dipeptidase, and finally acetylated by N-acetyltransferase to yield SFN-NAC.

To evaluate SFN’s potential as a mechano-modulator, a concise understanding of its basic pharmacology is necessary. SFN is generated upon hydrolysis of glucosinolates by the enzyme myrosinase, which is released when plant tissues are damaged or by gut microbiota [17] (Figure 1). The extraction of SFN employs various techniques, including solvent extraction, high hydrostatic pressure extraction, ultrasonic-assisted extraction, microwave-assisted extraction, solid-phase extraction, and pulsed electric field-assisted extraction [18,19,20]. Bioavailability is heavily influenced by food preparation; consuming raw broccoli (bioavailability ~37%) is far more effective than consuming cooked versions, where heat inactivates the myrosinase needed to convert glucosinolates to active SFN [21,22]. Its pharmacokinetics are characterized by rapid absorption and metabolism via the mercapturic acid pathway, which, along with relatively low oral bioavailability, has spurred the development of advanced delivery systems like nanoparticles to enhance its stability and efficacy [23,24]. For instance, SFN-loaded PEGylated Fe_3_O_4_@Au nanoparticles (NPs) have demonstrated a superior ability to induce apoptosis and necrosis compared to free SFN, while also potently suppressing the migration of human breast adenocarcinoma cells [25].

### 2.2. Molecular Reactivity and Target Engagement

The bioactivity of SFN originates from its electrophilic isothiocyanate group, which readily forms covalent adducts with cysteine thiols via Michael addition [23]. This direct chemical reactivity underpins its engagement with specific, primary protein targets. Key established direct targets include the sensor protein KEAP1 (leading to Nrf2 pathway activation), histone deacetylases (HDACs, resulting in histone hyperacetylation), and tubulin (causing cytoskeletal disruption) [26]. These initial, covalent modifications trigger a cascade of downstream cellular responses. Consequently, many critical nodes within mechanotransduction pathways—such as the transcriptional activity of YAP and β-catenin, or the activation of kinases like ROCK—are not directly alkylated by SFN. Instead, their activity is modulated indirectly as convergent consequences of upstream target engagement, alterations in redox balance, and integrated stress responses. Distinguishing this hierarchy from direct covalent targeting to downstream mechanosensitive signaling is crucial for a precise understanding of SFN’s mechanism as a multi-target “mechano-modulator.”

## 3. Chemopreventive Properties of SFN: A Biomechanical Perspective

Cancer chemoprevention aims to prevent, suppress, or reverse carcinogenesis using pharmacological or nutritional agents [27]. Epidemiological studies consistently show an inverse relationship between cruciferous vegetable consumption and cancer risk [28]. Preclinical evidence supports this; administering broccoli sprout extract or SFN prior to tumor cell inoculation in rats significantly suppresses subsequent tumor growth [29].

SFN’s chemopreventive mechanisms classically involve modulating phase I/II detoxification enzymes and inhibiting carcinogen-DNA adduct formation [30]. Upon exposure, carcinogens undergo sequential metabolic processes. Phase I metabolism activates procarcinogens by enhancing their reactivity and solubility [31]. It potently inhibits phase I enzymes like CYP1A2 and CYP3A4 and is a powerful inducer of phase II enzymes via Nrf2 activation—with a potency reported to be 14 times greater than quercetin [32,33]. This Nrf2-mediated upregulation of cytoprotective genes enhances cellular defense against carcinogens and oxidative stress [34].

Beyond biochemical detoxification, a novel perspective suggests SFN also acts through biomechanical pathways. Early stromal stiffening is a recognized feature of pre-malignancy [35]. By reinforcing cellular redox and structural homeostasis, SFN may help maintain normal tissue mechanics, counteracting biomechanical stresses that promote transformation [36]. This is supported by findings that SFN mitigates cellular dysfunction induced by mechanical stress in an Nrf2-dependent manner [37]. Thus, SFN provides a dual defense, bolstering both chemical detoxification and structural resilience.

## 4. Mechanobiological Mechanisms of SFN’s Anticancer Effect

### 4.1. Targeting the Tumor Cell Mechanostat

#### 4.1.1. Cell Cycle Arrest

**Figure 2:**
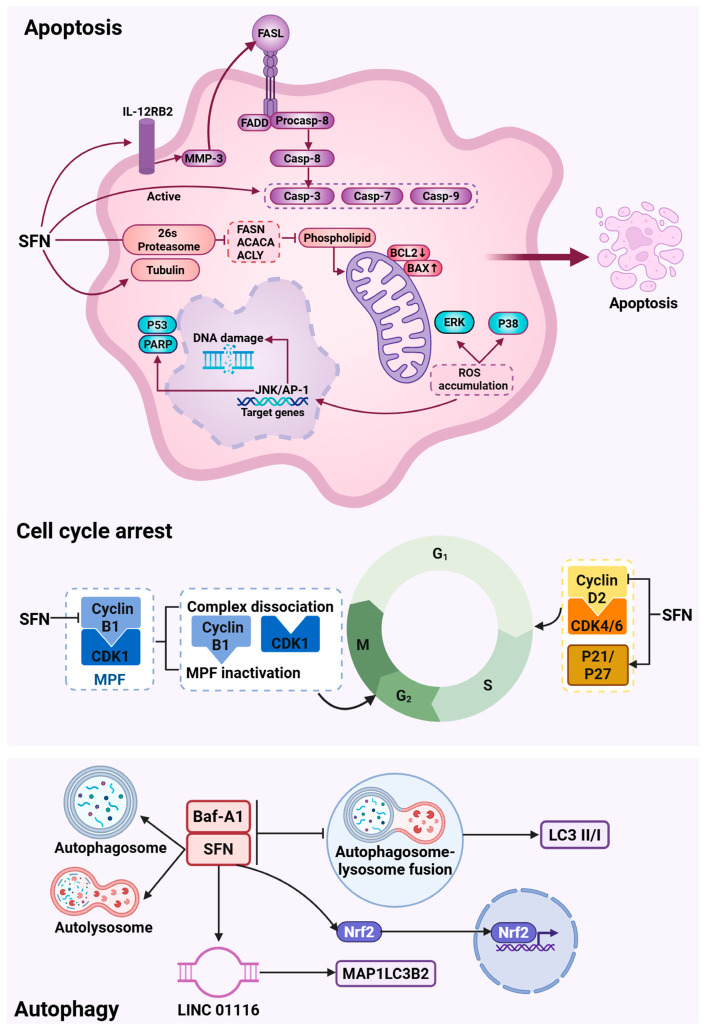
SFN induces tumor cell fate decisions via mechanosensitive pathways. SFN orchestrates cell cycle arrest, apoptosis, and autophagy, processes regulated by mechanical tension and adhesion. Cell cycle arrest: SFN disrupts key cyclin/CDK complexes and upregulates CDK inhibitors (p21, p27), arresting cells at G1/S and G2/M checkpoints. This may involve the inhibition of proliferation-associated mechanotransduction through FAK/Src and Rho/ROCK signaling. Apoptosis: SFN triggers both extrinsic and intrinsic mitochondrial pathways by altering the Bax/Bcl-2 ratio, inducing ROS, and activating executioner caspases. Concurrent proteasome inhibition disrupts the turnover of structural proteins, compounding biomechanical stress. Autophagy: SFN can induce a pro-survival autophagic response via Nrf2 activation, potentially to clear damaged cytoskeletal components. When autophagic flux is blocked, this process shifts toward promoting cell death.

Cell cycle progression is a mechanically sensitive process regulated by cyclins, CDKs, and their inhibitors [38,39]. SFN disrupts this machinery, inducing concentration-dependent G2/M phase arrest [40]. In classical Hodgkin lymphoma, it downregulates CCND2 while upregulating p21 and p27 [41]. A key mechanism is the dissociation of the cyclin B1/CDK1 (MPF) complex, critical for the G2/M transition, via downregulation of cyclin B1 [42]. This extensive disruption of cell cycle regulators may be linked to SFN’s inhibition of mechanosensitive pathways like FAK/Src and Rho/ROCK that coordinate proliferation with adhesive tension [43,44] (Figure 2).

#### 4.1.2. Apoptosis

SFN reactivates apoptotic pathways in cancer cells. It can induce the extrinsic pathway, for example, by activating the IL-12RB2/MMP-3/FasL cascade in myeloid-derived suppressor cells (MDSCs) [45]. More prominently, it triggers the intrinsic mitochondrial pathway by disrupting membrane potential, increasing the Bax/Bcl-2 ratio, and activating caspases-3, -7, and -9 [46]. This process involves ROS accumulation, which not only acts as an apoptotic trigger but also disrupts intracellular mechanical homeostasis by oxidizing cytoskeletal components, generating pro-apoptotic biomechanical stress [47,48]. SFN also targets the ubiquitin–proteasome system (UPS), inhibiting deubiquitinating enzymes USP14 and UCHL5 to enhance proteasomal degradation and induce apoptosis [49]. This UPS inhibition may also compromise the turnover of focal adhesion and cytoskeletal proteins, further destabilizing cellular mechanics [50] (Figure 2).

#### 4.1.3. Autophagy

Autophagy is a conserved catabolic process for maintaining homeostasis under stress, including mechanical strain [51,52]. SFN-induced autophagy, mediated through Nrf2 activation and HDAC6 inhibition [53,54], can be a pro-survival response aimed at clearing damaged mechanosensory components like the distorted cytoskeleton [55]. However, when this process is blocked (e.g., with bafilomycin A1), SFN’s effects lead to enhanced cell death, suggesting it pushes mechanical stress beyond a compensable threshold [56]. SFN regulates autophagy via multiple targets, including upregulating LINC01116 and MAP1LC3B2 in prostate cancer [57], promoting PTEN acetylation in breast cancer via HDAC6 inhibition [58], and upregulating HSP90AA1, UVRAG, and LAMP2 [59] (Figure 2).

### 4.2. Remodeling Malignant and Mechanical Phenotypes

#### 4.2.1. Epigenetic Modulation & Cancer Stem Cells

Epigenetic dysregulation is a hallmark of cancer [60]. SFN acts as a multi-target epigenetic modulator, inhibiting DNMTs and HDACs to influence DNA methylation and histone acetylation, and suppressing oncogenic lncRNAs like H19 [61,62,63,64]. Cancer stem cells (CSCs), drivers of tumor initiation and resistance, reside in specialized mechanical niches within the TME [65,66]. SFN effectively targets CSCs by inhibiting key stemness pathways. In colorectal cancer, it suppresses CSC properties via the ZNF217/Notch1 axis [67]. In lung cancer, it counteracts tobacco smoke-induced CSC phenotypes through the IL-6/ΔNp63α/Notch axis [68]. By disrupting these epigenetic and signaling networks—which are themselves sensitive to mechanical cues [69,70]—SFN likely alters the mechanosensitive transcriptional programs essential for CSC maintenance within stiff TME niches.

#### 4.2.2. Cell Migration and Invasion

**Figure 3:**
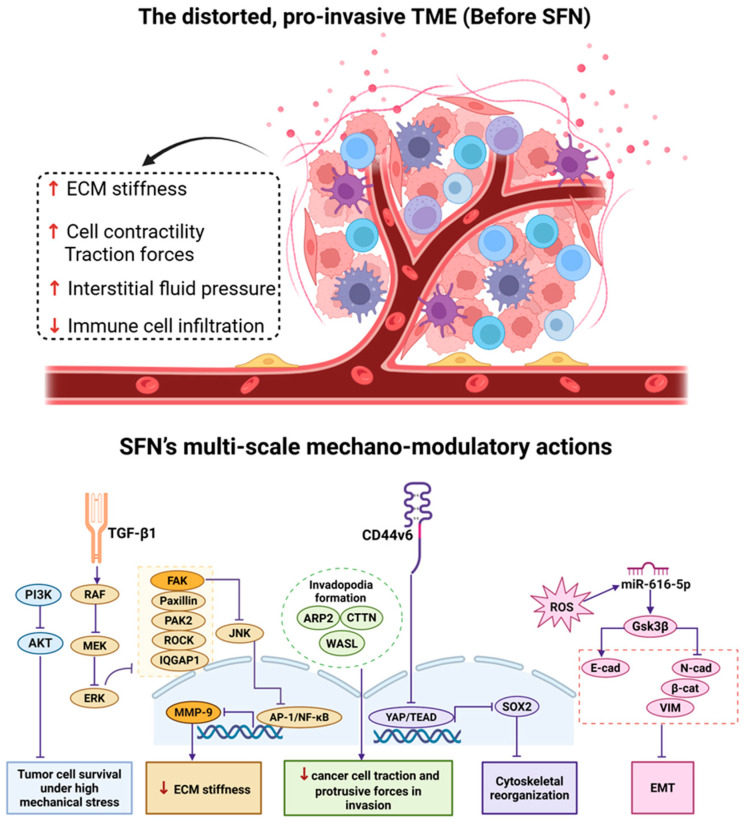
SFN regulates the pro-invasive tumor microenvironment through multi-scale mechanoregulation. Prior to SFN treatment: the tumor mechano-microenvironment is distorted and pro-invasive, characterized by elevated ECM stiffness, cell contractility/traction forces, interstitial fluid pressure, and reduced immune cell infiltration. SFN affects this pro-invasive landscape through multi-scale mechanomodulation: Inhibiting tumor cell survival under high mechanical stress (PI3K/AKT), disrupting cytoskeletal reorganization by suppressing TGF-β1/RAF/MEK/ERK signaling and downregulating stress fiber-associated proteins (Paxillin, FAK, ROCK) and CD44v6/YAP1/TEAD axis, impairing cancer cell traction and protrusive forces via the CTTN-ARP2/3 axis and WASL, reducing ECM stiffness by blocking FAK/JNK-mediated activation of AP-1 and NF-κB and repressing MMP expression, suppressing EMT by the miR-616-5p/GSK3β/β-catenin circuit. The arrows↑ represent increase and ↓ represent decrease.

Migration and invasion are hallmark characteristics of malignant tumors, enabling the multi-step process of metastasis [71]. SFN impedes metastasis by targeting multiple steps in the invasion cascade. SFN acts as a “cytoskeletal destabilizer.” It inhibits invadopodia formation by suppressing the cortactin-ARP2/3 axis and downregulating WASL (N-WASP) [72]. Concurrently, it impairs lamellipodia/filopodia integrity by inhibiting AKT1-mediated ATP production, crippling the energy supply for actomyosin contractility [73]. SFN suppresses ECM degradation by downregulating MMP-2 and MMP-9 and upregulating their inhibitor TIMP-3 [74]. It achieves this by blocking ROS-mediated activation of the transcription factors AP-1 and NF-κB [75,76], thereby preventing the mechanical feedback loop facilitated by matrix degradation and matrikine release [77]. SFN reverses EMT—a biomechanical reprogramming process—through multiple pathways: inhibiting Wnt/β-catenin in lung cancer [78], downregulating ZEB1 in breast cancer [79], suppressing the CD44v6/YAP1/TEAD axis in skin cancer [80], and inhibiting PI3K/AKT via ROS in thyroid cancer [81]. This promotes a reversion to a cohesive, mechanically stable epithelial phenotype [82,83]. In triple-negative breast cancer, SFN disrupts stress fibers—key drivers of contractility and migration—by suppressing TGF-β1-dependent RAF/MEK/ERK signaling [84]. This downregulates critical proteins like Paxillin, FAK, and ROCK, and involves direct binding to RAF [85,86]. This suggests a potential mechanobiological link between SFN and cell migration/invasion (Figure 3).

### 4.3. Modulation of Tumor Mechanical Microenvironment and Systemic Immunity

#### 4.3.1. Tumor Angiogenesis

SFN inhibits angiogenesis by suppressing key regulators like VEGF, HIF-1α, and c-Myc under hypoxic conditions [87,88]. Beyond starving the tumor, this anti-angiogenic action may promote “vascular normalization”—a transient restructuring of the abnormal, leaky tumor vasculature toward a more functional phenotype [89]. This process can reduce interstitial fluid pressure and mechanical stiffness, thereby improving drug perfusion and disrupting the pro-invasive mechanical niche [90].

#### 4.3.2. Immune Regulation in a Mechanically Hostile TME

**Figure 4:**
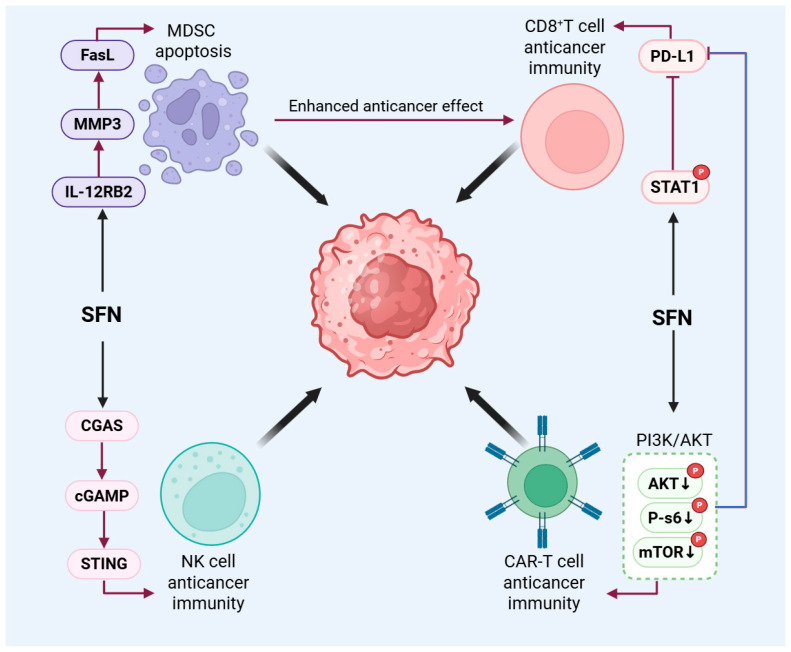
SFN remodels the immunosuppressive TME. SFN enhances antitumor immunity by targeting multiple immune cell types and modulating the physical TME. CD8^+^ T cell: SFN covalently modifies STAT1 to inhibit IFN-γ-induced PD-L1 expression on tumor cells, potentially improving T cell mechanosensing and function within a stiff ECM. MDSCs: SFN induces MDSC apoptosis via the IL-12RB2/MMP-3/FasL pathway, alleviating a major immunosuppressive barrier. By upregulating MMP-3 in CAFs, SFN promotes degradation of dense ECM components, reducing physical barriers to immune cell infiltration. CAR-T and NK Cells: SFN enhances CAR-T cell cytotoxicity and downregulates PD-1 via partial PI3K/AKT inhibition. It also potentiates NK cell activity, possibly through cGAS-STING activation linked to cytosolic DNA sensing.

The stiff, fibrotic ECM of the TME creates profound physical barriers to effective anti-tumor immunity [91]. Elevated tissue stiffness can impair cytotoxic T lymphocyte motility and infiltration [92], reduce the efficiency of immune synapse formation with cancer cells, and promote T cell exhaustion through altered mechanotransduction pathways [93]. This mechanically immunosuppressive landscape is maintained by stromal components like cancer-associated fibroblasts (CAFs) and suppressive immune cells such as myeloid-derived suppressor cells (MDSCs) [94,95]. SFN’s pleiotropic actions are positioned to alleviate these specific biomechanical constraints on immunity. By upregulating matrix metalloproteinase-3 (MMP3) in CAFs, SFN promotes degradation of dense ECM components like collagen III and fibronectin, potentially reducing the physical density that hinders immune cell trafficking [45]. SFN covalently modifies STAT1 to inhibit interferon-γ-induced PD-L1 expression on tumor cells. This downregulation of a key “brake” signal (PD-1/PD-L1) is hypothesized to improve immune synapse stability and counteract stiffness-associated exhaustion signaling, thereby enhancing CD8^+^ T cell activity [34]. SFN induces apoptosis in MDSCs via the IL-12RB2/MMP-3/FasL pathway, reducing a major cellular barrier to effective immunity. SFN modulates interactions between tumor-associated macrophages and the gut microbiota to intervene in colorectal cancer [96]. SFN improves chimeric antigen receptor T cell (CAR-T) function in solid tumor models by partially inhibiting the PI3K/AKT pathway, reducing PD-1 expression, and enhancing cytotoxic capacity [97]. Furthermore, SFN can enhance natural killer (NK) cell-mediated killing in classical Hodgkin lymphoma via activation of the cGAS-STING pathway [41]. Given that cytoskeletal rigidity can mediate cGAS-STING activation [90], SFN may initiate this immune response through its primary effects on cellular mechanics (Figure 4).

## 5. Conclusions

**Figure 5:**
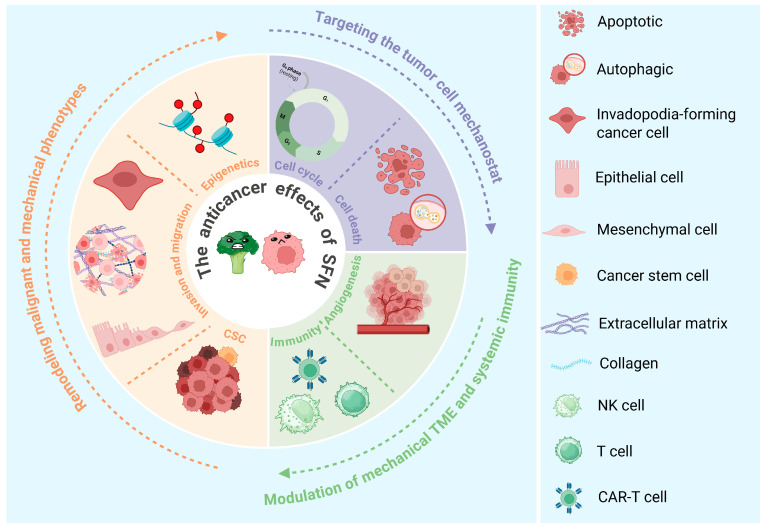
Sulforaphane as a multi-scale “mechano-modulator” in cancer. This schematic integrates SFN’s pleiotropic anticancer effects through the unifying lens of biomechanical normalization across scales. Molecular/cellular scale: SFN destabilizes the force-generating cytoskeleton, inducing cell cycle arrest and cell death (apoptosis and autophagy). Multicellular/tissue scale: SFN remodels the ECM, targets epigenetic modulation and CSCs in their niche, and may promote vascular normalization. Systemic scale: SFN enhances immune cell infiltration and function while inhibiting angiogenesis. These coordinated actions disrupt the distorted tumor mechano-environment, underpinning SFN’s efficacy against migration and invasion.

This review synthesizes evidence to position SFN as a “mechano-modulator” that normalizes the distorted biomechanical landscape of tumors. We hypothesize that its multifaceted actions—from inhibiting core mechano-signaling pathways (YAP/TEAD, Wnt/β-catenin) to destabilizing cellular invasion structures and remodeling the extracellular matrix—collectively counteract the physical drivers of malignancy (Figure 5).

To translate this compelling preclinical perspective into clinical reality, future research must adopt a dedicated mechano-informed approach. Key priorities include the following: (1) developing advanced formulations to improve SFN’s bioavailability and tumor-specific targeting; (2) employing biophysical tools (e.g., traction force microscopy) in 3D models to quantify SFN’s effects on cellular forces; (3) elucidating its impact on stroma-mediated tissue stiffness; (4) designing clinical trials that incorporate biomechanical biomarkers. Overcoming the challenges of dose-dependent complexity and the translational gap requires this focused strategy. Bridging molecular mechanism with tumor biophysics offers a promising path to harness SFN’s full potential as a pioneering therapeutic agent.

## Data Availability

The original contributions presented in this study are included in the article. Further inquiries can be directed to the corresponding author(s).
